# 6-Shogaol Inhibits Breast Cancer Cells and Stem Cell-Like Spheroids by Modulation of Notch Signaling Pathway and Induction of Autophagic Cell Death

**DOI:** 10.1371/journal.pone.0137614

**Published:** 2015-09-10

**Authors:** Anasuya Ray, Smreti Vasudevan, Suparna Sengupta

**Affiliations:** Division of Cancer Research, Rajiv Gandhi Centre for Biotechnology, Thiruvananthapuram - 695014, India; National Cancer Center, JAPAN

## Abstract

Cancer stem cells (CSCs) pose a serious obstacle to cancer therapy as they can be responsible for poor prognosis and tumour relapse. In this study, we have investigated inhibitory activity of the ginger-derived compound 6-shogaol against breast cancer cells both in monolayer and in cancer-stem cell-like spheroid culture. The spheroids were generated from adherent breast cancer cells. 6-shogaol was effective in killing both breast cancer monolayer cells and spheroids at doses that were not toxic to noncancerous cells. The percentages of CD44^+^CD24^-^/^low^ cells and the secondary sphere content were reduced drastically upon treatment with 6-shogaol confirming its action on CSCs. Treatment with 6-shogaol caused cytoplasmic vacuole formation and cleavage of microtubule associated protein Light Chain3 (LC3) in both monolayer and spheroid culture indicating that it induced autophagy. Kinetic analysis of the LC3 expression and a combination treatment with chloroquine revealed that the autophagic flux instigated cell death in 6-shogaol treated breast cancer cells in contrast to the autophagy inhibitor chloroquine. Furthermore, 6-shogaol-induced cell death got suppressed in the presence of chloroquine and a very low level of apoptosis was exhibited even after prolonged treatment of the compound, suggesting that autophagy is the major mode of cell death induced by 6-shogaol in breast cancer cells. 6-shogaol reduced the expression levels of Cleaved Notch1 and its target proteins Hes1 and Cyclin D1 in spheroids, and the reduction was further pronounced in the presence of a γ-secretase inhibitor. Secondary sphere formation in the presence of the inhibitor was also further reduced by 6-shogaol. Together, these results indicate that the inhibitory action of 6-shogaol on spheroid growth and sustainability is conferred through γ-secretase mediated down-regulation of Notch signaling. The efficacy of 6-shogaol in monolayer and cancer stem cell-like spheroids raise hope for its therapeutic benefit in breast cancer treatment.

## Introduction

Ginger (*Zingiber officinale*) is a well known herb consumed as a spice and food as well as widely used as herbal medicine for various ailments. A number of biologically active ingredients including gingerols and its various derivatives have been identified and synthesized from ginger in recent years. One important class of derivatives are shogaols that are primarily the dehydrated products of gingerols and are found exclusively in dried ginger. Among the shogaols, 6-shogaol has achieved a great deal of attention due to its potent anticancer activity against various cancer cells. It has been shown to induce mitotic arrest and reduce viability of gastric cancer cells [1]. Aberrant mitosis followed by apoptosis has also been found to be induced by 6-shogaol in HCT-116 colon cancer cells [2]. In human hepatoma p53 mutant Mahlavu subline, 6-shogaol induces apoptosis via oxidative stress pathway in a caspase dependent mechanism [3]. It has also been shown to induce autophagy in HNSCLC A-549 cells via inhibition of the AKT/mTOR pathway [4]. In another study, 6-shogaol has been reported to exhibit anti-invasive effects in breast cancer cells by reducing MMP-9 expression through NF-κB activation [5]. Recently, PPAR-γ dependent apoptosis in MCF-7 and HT-29 cells by 6-shogaol has also been reported [6]. Additionally, recent studies have implicated microtubule as a possible target of 6-shogaol as it interacts with the sulphydryl groups of cysteines in tubulin through its side chain containing the α, β unsaturated carbonyl moiety [7]. All these studies place 6-shogaol as a promising agent to be studied further in view of its future therapeutic potential in cancer therapy.

Cancer stem cells play a very important role in cancer development and progression. The concept of stem cell origin of cancer has been supported by observations that certain subpopulations (only 0.2–1%) of cancer cells have stem cell-like properties, such as the ability to self renew, continuous differentiation and an overall innate resistance to conventional chemotherapeutic agents [8]. These chemo-resistant, self-renewing, tumorigenic sub-population of cells defined as cancer stem cells (CSCs) play crucial roles in cancer recurrence. CSCs have been identified in various solid tumors including breast, ovarian, head and neck, pancreas, and colon cancer [9, 10]. Earlier studies demonstrated that the signaling pathways such as Wnt/β-catenin, Notch and Hedgehog pathways regulate the growth of cancer stem cells [11, 12]. Therefore, targeting these pathways is considered to be a useful strategy to inhibit cancer stem cell regeneration.

Although, CSCs are present in a very small percentage in the total tumour, methods have been developed to grow them in large population in *ex vivo*. In appropriate growth conditions, cancer cells can be made to grow in the form of spheroids. These spheroid-forming cells exhibit altered cell surface markers when compared to cells grown in monolayer culture and have been shown to possess stem-cell like properties [10, 13]. These spheroids have been used in a number of studies to determine the *in vitro* and *in vivo* characteristics of cancer stem cells as well as to assess the inhibitory activity of cytotoxic compounds against cancer stem cells [11, 14, 15].

Several *in vitro* studies have shown that cancer stem cells are resistant to conventional chemotherapeutic drugs [8, 16]. Interestingly, a number of dietary compounds like curcumin [14], piperine [14], sulforaphane [17] have recently been identified to target CSCs. However, various factors such as toxicity, weak dose response etc. largely limit their application. Since 6-shogaol has been reported as a potent anticancer agent against various cancer cells, we have investigated its inhibitory effect on breast cancer cells and cancer stem cell-like spheroids. Here we demonstrate that 6-shogaol shows anti-proliferative activity against breast cancer cells and spheroids and suppresses the size and colony forming ability of spheroids by altering the Notch signaling pathway. Investigation of the death mechanism shows that autophagy is a predominant mode of cell death caused by 6-shogaol in breast cancer cells.

## Materials and Methods

### Materials

6-shogaol (≥90%), Taxol (≥95%), and DAPT (N-[N-(3,5-difluorophenacetyl)-l-alanyl]-S-phenylglycine t-butyl ester) (≥98%) were purchased from Sigma. Chloroquine (CQ) was from Molecular Probes, Invitrogen. Fluoromount G was procured from Electron Microscopy Sciences. DAPI (4',6-Diamidino-2-Phenylindole), Giemsa and other fine chemicals were from Sigma. Chemiluminescent western blotting detection system was from Thermo Scientific. FITC Annexin V Apoptosis Detection kit was purchased from BD Pharmingen (Cat # 556547). Ultra low attachment plates were obtained from Corning, USA and MEBM (Mammary Epithelial Basal Media) was procured from Lonza, USA.

### Antibodies

PE (Phycoerythrin)-conjugated CD44 (555749) and FITC (Fluorescein Isothiocyanate)-conjugated CD24 (555573) antibodies were purchased from BD Biosciences. Antibodies for Cleaved Notch1 (4147S) and Cyclin D1 (IMG-6583A) were procured from Cell Signalling Technology and Imgenex respectively. Antibodies for PARP were from Cell Signaling Technology (CST-9544) and Santa Cruz Biotechnology (sc-7150); Bcl-2 (sc-7382), Bax (sc-7480), β-actin (sc-47778) and Hes1 (sc-166378) were from Santa Cruz Biotechonology. Primary antibody for LC3A/B (Light Chain 3) (ab-173752) was obtained from Abcam or from Cell Signaling Technology (CST-12741). Anti-mouse and anti-rabbit HRP were purchased from Sigma. Anti-rabbit alexa 488 was from Molecular Probes, USA.

### Cell lines

Human metastatic breast adenocarcinoma cell lines MCF-7 and MDA-MB-231 were obtained from National Cancer Institute, USA (ATCC# HTB-22 and ATCC# HTB-26 respectively). Human embryonic kidney cell line HEK 293 (ATCC# CRL-1573.3) was obtained from ATCC. The human immortal keratinocyte cell line HaCaT [18] was obtained from the national repository of National Centre for Cell Sciences, Pune, India. Frozen stocks of cells from the reference stock were made within passage 3 and stored in liquid nitrogen. For experiments, cells were used within 2 months of revival.

### Cell culture

MCF-7 cells, HEK 293 and HaCaT cells were maintained in DMEM/F12 supplemented with 1% sodium pyruvate, 0.2% non-essential amino acids, 1% penicillin-streptomycin and 10% FBS in a humidified atmosphere containing 5% CO_2_ at 37°C. MDA-MB-231 cells were maintained in Leibovitz L-15 media, 1% penicillin-streptomycin and 10% FBS under the same condition. For spheroid culture, Mammary Epithelial Basal Media (MEBM) was supplemented with 0.001% Epidermal Growth Factor (EGF), 0.001% Insulin, 0.004% Bovine pituitary extract (BPE) and 0.001% Hydrocortisone. 0.001% Gentamicin sulphate and amphotericin-B were added as antibiotic-antimycotic [hereafter termed as MEGM (Mammary Epithelial Growth Media)]. MCF-7 and MDA-MB-231 cells were seeded in ultra low attachment plates using MEGM as culture medium. After three days of seeding, cells forming spherical clusters were considered as stable spheroids and used for further experiments.

### Immunocytochemistry

To check cell surface marker expressions, monolayer MCF-7 cells were grown on coverslips. Cells were fixed in Methanol-EDTA (1 mM EDTA) for 10 minutes and rehydrated in PBS. After blocking, cells were incubated with PE-conjugated CD44 antibody (1:50) for 3 hours at room temperature and stained with DAPI (1 μg/ml). MCF-7 spheres in suspension were fixed in 4% paraformaldehyde for 10 minutes at -20°C and permeabilised in 0.1% triton X-100 for 10 minutes in PBST (PBS with 0.05% Tween-20). Cells were then processed as mentioned above. Thereafter, cells were mounted on slides with Fluoromount G for imaging.

To check for the induction of autophagy, the MCF-7 monolayer cells were seeded on coverslips. 70–80% confluent cells were treated with 6-shogaol or chloroquine. The cells were then fixed, permeabilized and blocked as mentioned earlier. Thereafter the cells were incubated overnight with LC3A/B primary antibody (1:100), followed by anti-rabbit alexa 488 secondary antibody (1:200) for one hour. DAPI staining and mounting were done as above. Visualization was done under 60X oil immersion objective in a confocal microscope (Nikon Eclipse Ti (A1R) Japan). Z-stacking was done wherever necessary.

### Flow cytometric analysis of cell surface markers

Monolayer cells or spheroids (with or without treatment of 6-shogaol) were dispersed by trypsin-EDTA. Cells were then pelleted down by centrifugation at 1600 rpm for 5 minutes at 4°C and resuspended in 1 ml of DMEM containing 2% FBS. Fluorescein isothiocyanate (FITC)-conjugated CD24 monoclonal antibody and phycoerythrin (PE)-conjugated CD44 monoclonal antibody (BD Biosciences) were added in 1:100 dilution and incubated on ice for 45 minutes in dark. The cells were then analyzed by flow cytometer (BD FACS Aria II) for the expression of CD24 and CD44.

### Cytotoxicity assay

Monolayer cells (5×10^3^cells/well) were seeded in quadruplicates in 96 well plates and were incubated with varying concentrations of 6-shogaol (1–100 μM), taxol (0.2–100 nM) or curcumin (4–80 μM). The effect of the drugs on the viability of all the cell lines was quantified by MTT (3-[4,5-dimethylthiazol-2-yl]-2,5 diphenyltetrazolium bromide) assay at different time points after drug treatment using a Biorad Plate reader [19]. The IC_50_ values were calculated from the plot of percentage cell viability versus concentration of drug using the nonlinear regression programme of Origin.

To check the effect of different drugs on the viability of spheroids, cells were seeded at 2.5×10^4^ cells/well in 24-well ultra-low attachment plates and grown as spheroids. After 3 days, 6-shogaol (1–100 μM) or taxol (5 nM-50 μM) or curcumin (2–50 μM) was added to the wells and MTT assay was performed as explained above. Standard deviation was calculated from three independent experiments and the data were represented as the average of three experiments.

### Cytotoxicity assay with Chloroquine

Monolayer MCF-7 cells were incubated for 48 hours with either chloroquine (CQ) or 6-shogaol individually or in combination and cell viability was checked by MTT assay. Chloroquine was added 1 hour prior to 6-shogaol addition.

### Cell cycle analysis

Cell cycle distribution of cells treated with 6-shogaol was analyzed by flow cytometric measurement of cellular DNA content. MCF-7 cells (5×10^5^ cells/well) were subjected to 6-shogaol treatment for various time points. Culture media containing detached cells were collected and attached cells were trypsinized. Cells were pelleted, fixed in 70% (v/v) ethanol and stored at 4°C. In case of spheroids, cells were treated with different concentrations of 6-shogaol for 48 hours and fixed for further analysis. Before analysis, cells were re-washed with PBS and incubated with 10 μg/ml RNaseA and 400 μg/ml propidium iodide for 30 minutes at 37°C. Data were analyzed using FACS Diva software.

### Formation of primary and secondary sphere

5000 cells/well were seeded in ultra low attachment plates. After 3 days of seeding, the spheres formed were kept with different concentrations of 6-shogaol and/or 25 μM DAPT for 7 days and the number of spheres were counted under the microscope. These primary spheres were dispersed and kept in fresh media without any treatment and counted again after 7 days to check the ability to regrow into secondary spheres. Control primary spheres were also processed similarly for comparison. Spheres were counted in Olympus IX71 microscope using 10X objective.

### Western blot experiments

Whole cell lysates were prepared using phospho-lysis buffer containing 10% NP-40, 10% glycerol, 137 mM NaCl, 20 mM Tris-HCl (pH 7.4), 20 mM NaF, 1 mM sodium pyrophosphate, 1 mM sodium orthovanadate, 1% Triton X-100, and 5 mM PMSF in presence of protease inhibitor cocktail (10 μl/ml). Lysates were placed into ice for 1 hour to ensure complete lysis followed by centrifugation at 14,000 g for 10 minutes and the supernatant was collected. Proteins were subjected to SDS-PAGE and transferred to polyvinylidene fluoride membrane. The membranes were blocked and incubated overnight with primary antibodies at 4°C followed by incubation with horseradish peroxidase tagged secondary antibodies. The blots were developed using ECL reagent. The primary antibodies were used in the following dilutions: Cleaved Notch1 (1:2000), Hes1 (1:200), Cyclin D1 (1:400), PARP (1:500 for sc) and (1:1000 for CST), LC3A/B (1:500), Bcl-2 (1:1000), Bax (1:1000) and β-actin (1:2000). β-actin was used as loading controls.

### DAPI staining for studying apoptosis

Cells were seeded on cover slips. 70–80% confluent cells were treated with 6-shogaol (15 μM) for 48 hours and 72 hours. Cells were washed with PBS, fixed with Methanol-EDTA for 10 minutes at -20°C followed by rehydration with PBS at room temperature and incubated with 1 μg/ml DAPI. Mounted coverslips were visualised at 40X magnification under fluorescence microscope (Olympus IX71). Apoptotic cells were counted from five different fields.

### AnnexinV/PI staining for studying apoptosis

Apoptosis was quantified by using a FITC Annexin V Apoptosis Detection kit according to manufacturer’s instructions. MCF-7 cells were treated with 15 μM and 25 μM of 6-shogaol for 24, 48 and 72 hours, respectively. Attached and floating cells were collected and analyzed by a flow cytometer (BD FACS Aria II).

### Giemsa staining

Cells were seeded in 35 mm glass bottom dishes. 70–80% confluent cells were treated with 6-shogaol (15 μM) for 48 hours. Both control and treated cells were washed with PBS, fixed in methanol-EDTA, rehydrated in PBS and incubated for 1 hour in Giemsa stain. Cells were air dried and imaged in Olympus IX71 fluorescent microscope under 40X objective.

### Statistical Analysis

Densitometry was conducted for all the western blots using BD Quantity One software (Bio-Rad). Normalisation was done according to the loading control and fold change was calculated with respect to the control. For graphs, results are depicted as mean ± standard error of mean or standard deviation (calculated from two or more experiments). The p-values were obtained using Student’s unpaired *t*-test and p < 0.05 was considered to be statistically significant.

## Results

6-shogaol has been shown to exert inhibitory effect on various cancer cell lines and animal disease models [20, 21]. To check whether 6-shogaol is effective on breast cancer stem cells, we generated model stem cell-like spheroids from two types of breast cancer cell lines, ER/PR positive MCF-7 and triple negative MDA-MB-231. The cells were grown in low attachment plates with conditioned media that promote non-adherent growth. Under these conditions, the cells became subsequently organized as clusters of spherical cells. After 3 days of growth, the clusters were considered as stable spheroids. All the further experiments were performed under these conditions.

### Expression of CD44/CD24 in spheroids

**Fig 1:**
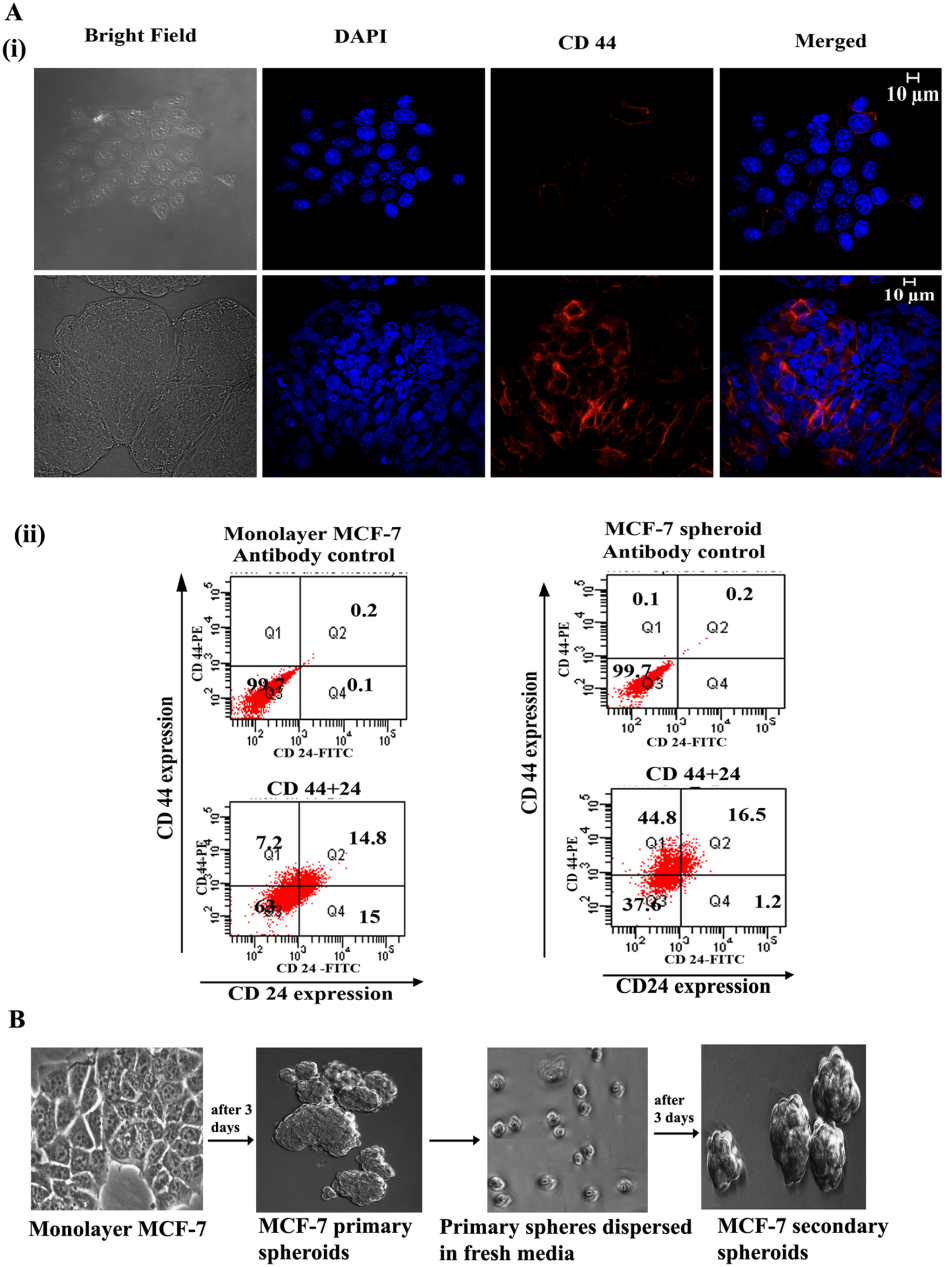
Characterization of breast cancer stem cell like spheroids. (A) Expression of cell surface markers CD44 and CD24 in MCF-7 monolayer and spheroids. (i) Immunofluorescence images showing expression of CD44 in MCF-7 monolayer cells (upper panel) and in MCF-7 spheroid cells (lower panel). Cells were stained with PE-conjugated CD44 antibody and imaged as described in the methods section. (ii) Flow cytometric analyses of CD44/CD24 in monolayer MCF-7 cells (left panel) and in MCF-7 spheroids (right panel). (B) Formation of primary and secondary spheres by MCF7 cells. Image was taken in 10X in an Olympus IX71 microscope.

Differential expression of cell surface markers between monolayer cells and CSCs help to select CSCs within the tumour. CD44 and CD24 are cell surface glycoproteins that play a major role in both cell adhesion and migration. Earlier studies have shown that breast cancer cells contain stem-cell like cells exhibiting CD44^+^CD24^-^/^low^ marker expression and these cells possess more than 50 fold increased tumorigenic capacity when compared to the other cells of tumour mass [13, 16, 22]. It has been reported that cultured mammospheres also exhibit stem cell like properties with characteristic CD44^+^CD24^-^/^low^ expression [23, 24]. We therefore checked whether the spheroids generated by us possessed this phenotype. Immunofluorescence images showed that expression of CD44 [Fig 1A(i)] was significantly higher in the spheroids generated from MCF-7 cells than in the monolayer MCF-7 cells. Concomitant with this, flow cytometric analysis also showed significantly high CD44 and low CD24 expression in spheroid forming cells than in monolayer cells [Fig 1A(ii)].

Further, it has been demonstrated that stem cell-like spheroids are capable of generating next generation (secondary) spheres and possess the ability to differentiate along multiple lineages [25]. Spheroids generated by us also exhibited the property of formation of secondary spheres as shown in Fig 1B. These spheroids were used for further experiments to investigate the effect of 6-shogaol on cancer stem-like cells.

### Cytotoxicity study of 6-shogaol against monolayer and spheroid cells

The anti-proliferative activities of 6-shogaol on monolayer cells and spheroids were quantitated by MTT assay in two cell lines, MCF-7 and MDA-MB-231. Cells were treated with increasing concentrations of 6-shogaol for 48 hours and then the cell viability was measured. The IC_50_ values are shown in Table 1. Taxol, a drug widely used in breast cancer treatment, and curcumin, a compound earlier shown to be effective in inhibiting cancer stem-like spheroids [14], were used for comparison. The results showed that for both the cell types, 6-shogaol was effective in spheroids at concentrations that were 5 or 2 fold higher than the effective inhibitory concentrations in monolayer cells. In contrast, taxol, even though was highly active in monolayer cells, did not show activity against the spheroids even at 10000 fold higher concentration compared to 6-shogaol (Table 1). Curcumin was also found to be effective against MCF-7 spheroids as reported earlier [14]. The results also showed that 6-shogaol was ~3 fold more potent against MDA-MB-231 spheroids than against MCF-7 spheroids. The inhibitory effect of all the three compounds on non-cancerous cell lines, HEK 293 and HaCaT were also tested (Table 1). The IC_50_ data showed that the noncancerous cells tolerated significantly higher doses of 6-shogaol compared to the cancer cells in both monolayer and spheroid culture conditions. Non-cancerous cells were quite resistant to taxol when compared with its efficacy in the monolayer breast cancer cells. In contrast, curcumin did not exhibit any resistance to noncancerous cells as compared to its action on breast cancer cells and spheroids. Thus the action of 6-shogaol on breast cancer spheroids is superior to taxol or curcumin considering the fact that it inhibits spheroids at concentrations which are safe to non cancerous cells.

**Table 1:** Cytotoxic activity of 6-shogaol in breast cancer cells and spheroids and in noncancerous cells. Different concentrations of drugs were added one day and three days after seeding for monolayer and spheroids respectively. IC_50_ was determined from MTT assay results after 48 hours using the nonlinear regression programme of Origin. Standard deviations from three different experiments are shown.

	6- shogaol [IC_50_]	Taxol [IC_50_]	Curcumin [IC_50_]
MCF-7 monolayer cells	7.94±0.57 μM	4.92±0.71 nM	37.14±0.85 μM
MCF-7 spheroid	39.52±0.62 μM	>50 μM	7.27±0.54 μM
MDA-MB-231 monolayer cells	5.67±0.73 μM	2.86 ±0.66 nM	Not checked
MDA MB-231 spheroid	11.18±0.83 μM	>20 μM	Not checked
HaCaT cells	103.84±0.67 μM	42.67±0.75 nM	10.4±0.87 μM
HEK 293 cells	69.97±0.52 μM	103.79±0.48 nM	23.06±0.68 μM

**Fig 2:**
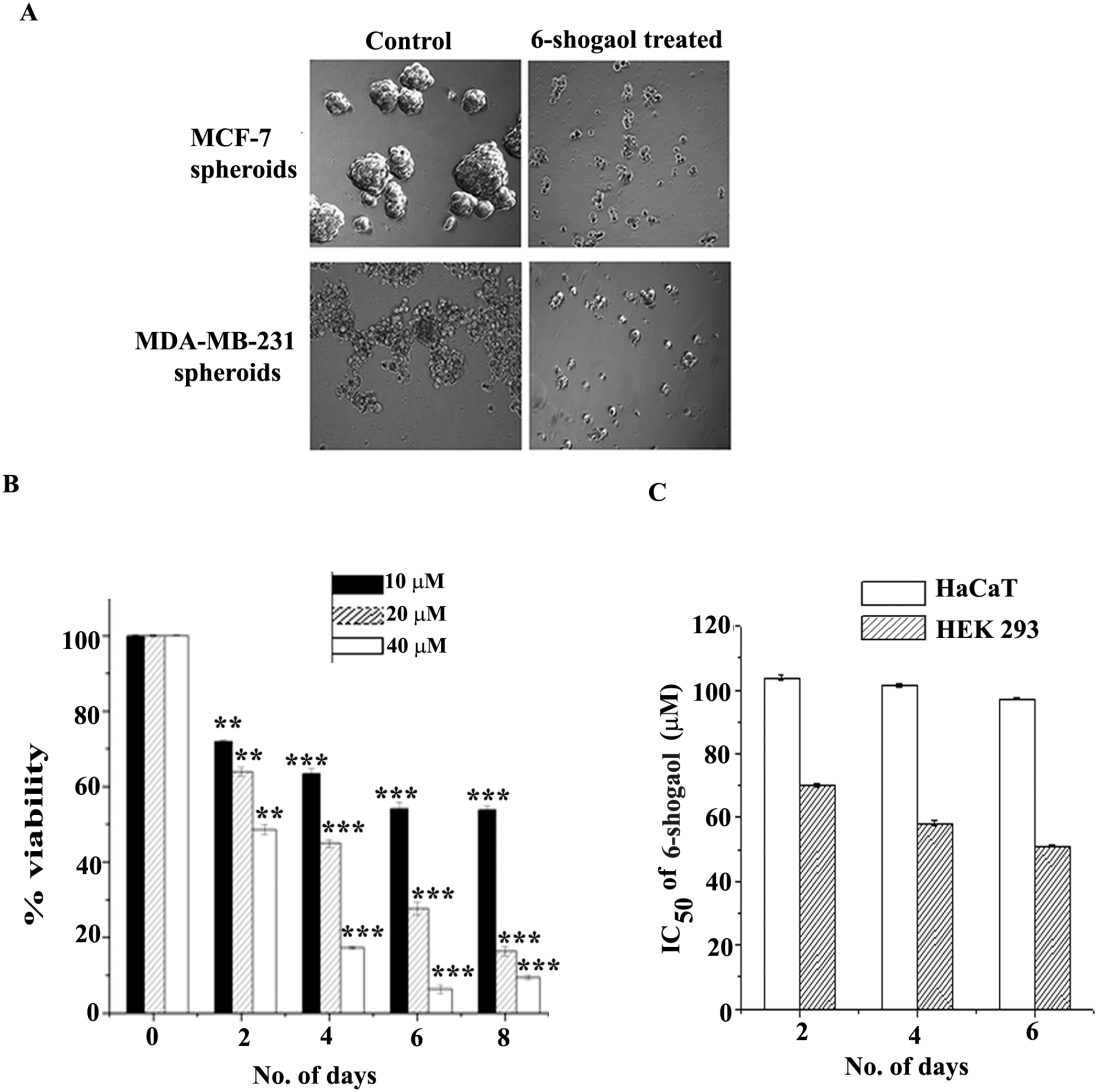
Inhibitory effect of 6-shogaol on MCF-7 and MDA-MB-231 breast cancer spheroids. (A): Breast cancer spheroids with or without treatment. MCF-7 spheroids with 40 μM of 6-shogaol (upper panel); MDA-MB-231 spheroids with 11 μM 6-shogaol (lower panel). (B): Prolonged effect of different concentrations of 6-shogaol on MCF-7 spheroids. The error bars represent the standard error of mean from three different experiments. *** refers p ≤ 0.001; ** refers p ≤ 0.005; * refers p ≤ 0.05. (C): Prolonged effect of 6-shogaol on noncancerous cell lines HEK 293 and HaCaT. The error bars represent the standard error of mean from three different experiments. The differences among the different treatment periods up to 6 days were found to be insignificant.

The changes in size of the spheroids generated from MCF-7 and MDA-MB-231 cells after treatment with 6-shogaol are shown (Fig 2A). Interestingly, MDA-MB-231 cells formed relatively loose and less rounded spheroids than those of MCF-7 cells, which was probably the reason for better penetration and more effectiveness of 6-shogaol in MDA-MB-231 spheroids than MCF-7 spheroids. This also indicated that 6-shogaol might need longer time to penetrate into MCF-7 spheroids. We thus investigated the effect of prolonged treatment of low dose of 6-shogaol (range of IC_50_ and below) on the viability of MCF-7 spheroids. The experiments were performed under three sets of 6-shogaol concentrations, 40 μM (IC_50_), 20 μM (½ IC_50_), and 10 μM (¼ IC_50_). The spheroids formed after 3 days of seeding were treated with 6-shogaol and further the effect on the cell viability were measured after every two days till the 8th day. 6-shogaol reduced the viability of the spheroids in a time dependent manner (Fig 2B). On day 4, 10 μM, 20 μM and 40 μM 6-shogaol inhibited cell viability by ~35%, 56%, and 83% respectively. After 6 days, 10 μM 6-shogaol treatment resulted in 46% inhibition. Likewise, to check the effect of prolonged treatment of 6-shogaol on non-cancerous cells, HEK 293 and HaCaT cells were treated with 6-shogaol for 6 days. However, in these cells the IC_50_ values showed no significant decrease up to 6 days when compared with 2 days of treatment. The results showed that both the cell lines could tolerate considerably high concentration of 6-shogaol till day 6 (Fig 2C).

### Cell cycle analysis of 6-shogaol treated cells

**Fig 3:**
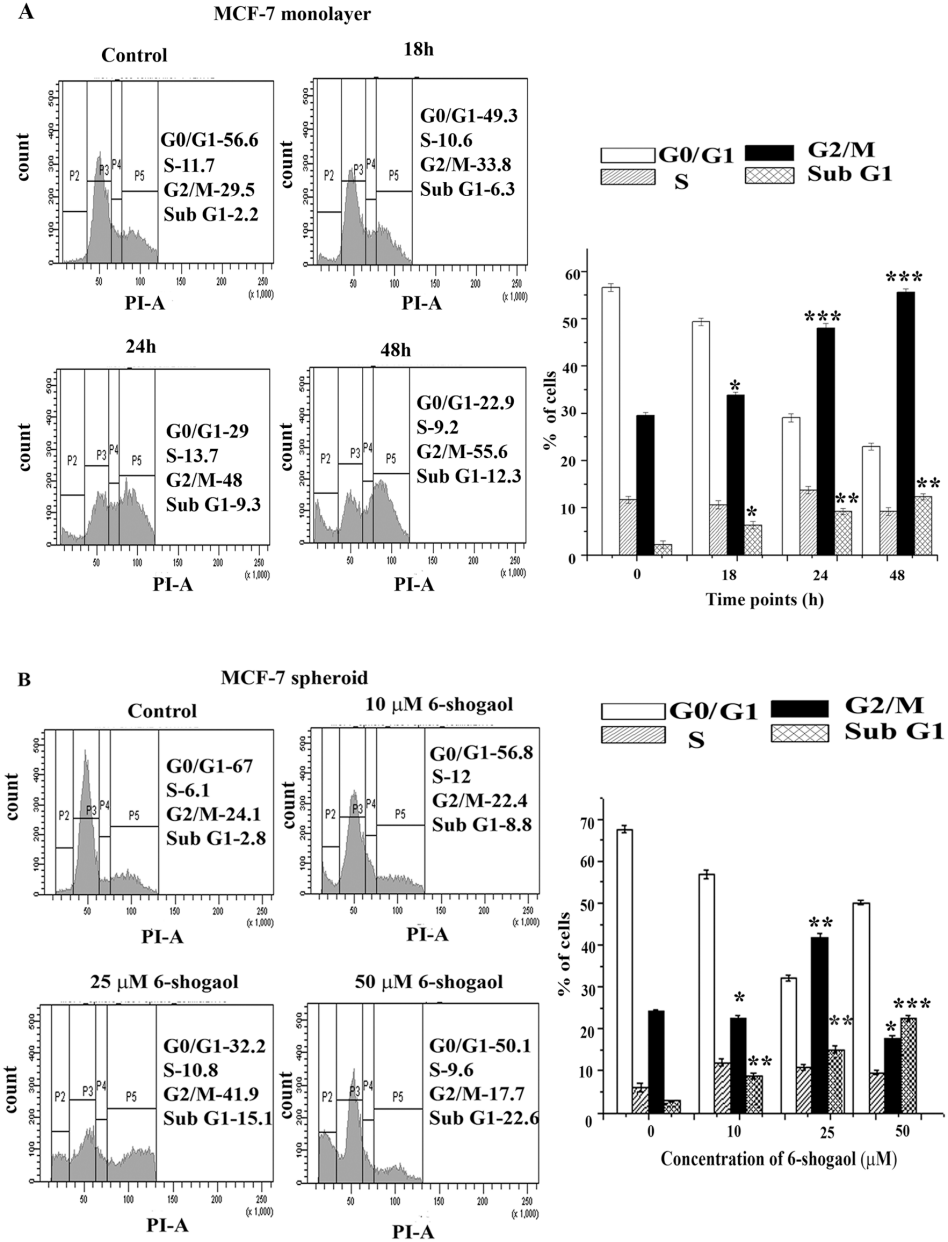
Effect of 6-shogaol on cell cycle of MCF-7 cells / spheroids. (A): Cell cycle analysis of MCF-7 cells treated with 16 μM 6-shogaol (2×IC_50_) for different time points. (B): Cell cycle analysis of MCF-7 spheroid cells with different concentrations of 6-shogaol for 48 hours. The histogram is a representative of three independent experiments for both monolayer and spheroid cells. Bar graph represents percentage of cells in different phases of cell cycle. Error bars represent standard error of mean and have been calculated from three different experiments. *** denotes p ≤ 0.001; ** denotes p ≤ 0.005 and * denotes p ≤ 0.05.

To understand the mechanism of action of 6-shogaol, its effect on cell cycle progression was analysed by flow-cytometry. Monolayer cells were treated with 16 μM (2×IC_50_) of 6-shogaol for different time points. As evident from DNA content measurement, 6-shogaol induced cell cycle arrest at G2/M phase (Fig 3A). After 24 and 48 hours of treatment, percentage of cells in the G2/M phase increased from 29% (control) to 48% and 55%, respectively.

Effect of 6-shogaol on cell cycle of MCF-7 spheroid cells was also analysed by flow cytometry. Spheroids were treated with different concentrations (0–50 μM) of 6-shogaol for 48 hours. 25 μM of 6-shogaol treatment exhibited 42% G2/M arrest as compared with 24% in control. However, G2/M percentage was found to decrease when treated with 50 μM 6-shogaol. It was observed that the cells in the Sub G1 phase increased gradually with increased 6-shogaol concentration (Fig 3B), indicating that like in monolayer cells, 6-shogaol treatment led to the spheroid cell death in a concentration dependent manner.

### Cell death mechanism induced by 6-shogaol

Next, we examined whether the cell death induced by 6-shogaol in breast cancer cells was due to apoptosis. Apoptotic cell death is characterized by hallmark changes like chromatin condensation, membrane blebbing, DNA fragmentation as well as cleavage of the DNA repair protein PARP (Poly-ADP-Ribose Polymerase).

**Fig 4:**
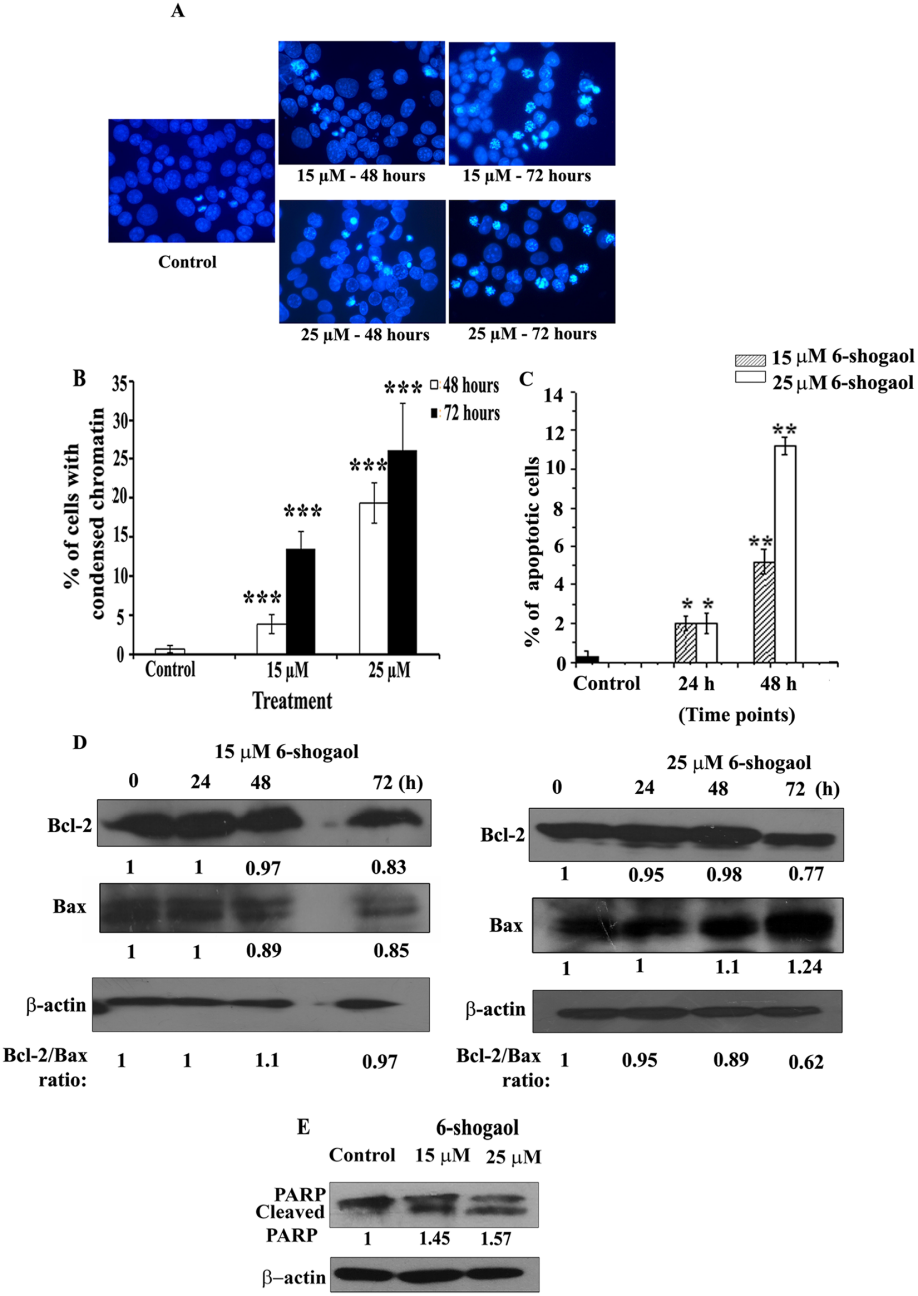
Induction of apoptosis by 6-shogaol. (A): DAPI staining showing condensed nuclei in MCF-7 cells in presence of the indicated concentrations of 6-shogaol after 48 hours and 72 hours. (B): Graph quantitates the percentage of apoptotic cells for each treatment and time points. The results are representative of three independent experiments. The error bars represent standard deviation. *** denotes p ≤ 0.001 and ** denotes p ≤ 0.005. (C): Graph represents the percentage of Annexin V positive cells for each 6-shogaol treatment and time points. The error bars represent standard deviation. ** refers p ≤ 0.005; * refers p ≤ 0.05. (D): Western blot showing expression of Bcl-2 and Bax upon 6-shogaol treatment. (E): Western blot showing reduction in the mother band of 116 kDa and formation of 85 kDa cleaved band of PARP upon treatment with the indicated concentrations of 6-shogaol after 72 hours. Blot was probed with sc-7150 PARP antibody. β-actin is the loading control for all the blots. All the blots are representative of three independent experiments.

As measured by chromatin condensation in MCF-7 cells, only 19% and 28% apoptosis was observed after 48 and 72 hours of 25 μM 6-shogaol treatment, respectively (Fig 4A and 4B). We also quantified 6-shogaol induced apoptosis by other apoptotic markers like staining of phosphatidyl serine by annexin V-FITC and Bcl-2/Bax ratio. Annexin V/PI flow-cytometric assay detected only 11% of early apoptotic cells (Annexin positive/PI negative) after 48 hours of 25 μM 6-shogaol treatment on MCF-7 cells (Fig 4C and S1A Fig), even though both 15 μM and 25 μM 6-shogaol treatments resulted in a large amount of cell death (consisting of late apoptotic (Annexin positive/PI positive) cells and cells dead by other mechanisms (PI positive/Annexin negative) after 72 hours (S1A Fig). Bcl-2/Bax ratio also showed decrease only after 72 hours treatment of very high concentration (25 μM) of 6-shogaol (Fig 4D). Further, western blotting detected PARP cleavage in MCF-7 cells treated with 15 μM and 25 μM of 6-shogaol only after 72 hours of treatment (Fig 4E and S1B Fig). However, in breast cancer spheroids, we were not able to detect appreciable amount of apoptosis after 40 μM of 6-shogaol treatment even upto 72 or 96 hours. Further higher concentrations could not be tried due to massive cell loss. These results indicated that even after prolonged treatment, 6-shogaol induced apoptosis only partially in MCF-7 cells. We then examined whether any other cell death mechanism was induced by 6-shogaol.

Several studies have reported the activation of autophagy in response to various anticancer agents. Autophagy is characterized by massive degradation of portions of cytoplasm and intracellular organelles by forming Acidic Vesicular Organelles (AVO). Recruitment of the microtubule associated protein Light Chain3 (LC3) to autophagosomes as LC3-I and subsequent formation of LC3-II upon lipidation are also considered as early events of autophagy [26]. Autophagy is a complex cellular process which can be both pro-survival and pro-death. Several stress conditions may induce this evolutionarily conserved process to give survival advantage to the cells and thus, the autophagy inhibitors may cause apoptosis by inhibiting these survival signals [26, 27]. However, it has also been found that several external agents use autophagy as an alternate mode of cell death in absence of prominent apoptosis [4, 28].

**Fig 5:**
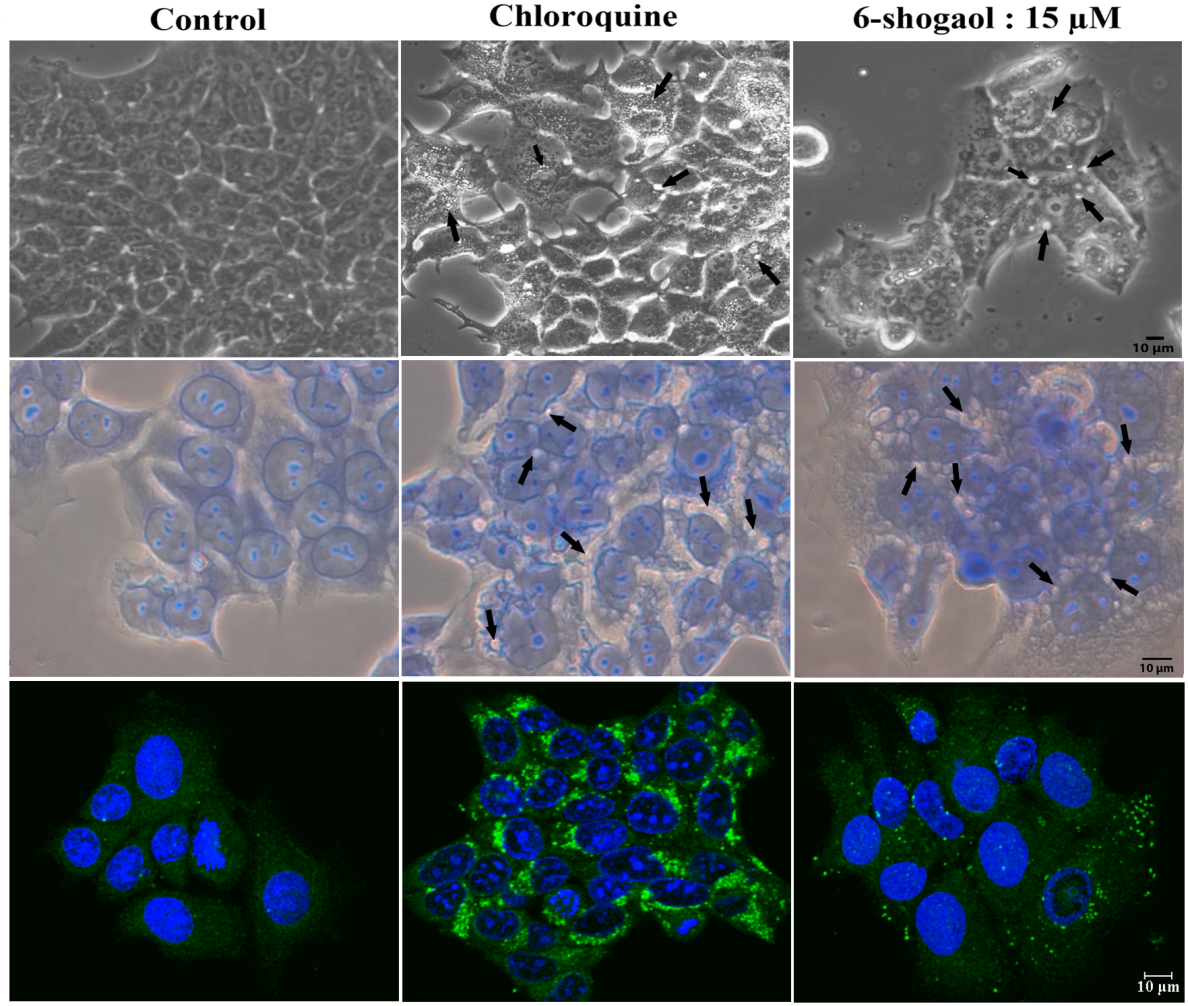
Induction of autophagy by 6-shogaol. MCF-7 cells were treated with 6-shogaol (15 μM) or chloroquine (50 μM) for 48 hours. Top panel: Bright field image (20X) of control and treated cells. Arrow heads indicates cytoplasmic vacuolization. Middle panel: Giemsa stained cells where arrows point towards vacuoles. Bottom panel: Cells were stained with LC3A/B primary antibody and immunocytochemistry was conducted as described in the Methods section. Green punctate staining in the 6-shogaol or chloroquine treated cells shows the localization of LC3 in autophagosomes. Cell nuclei are labelled blue (DAPI). The scale bar represents 10 μm for all the three panels.

As shown in Fig 5, 48 hours treatment of 6-shogaol induced a large number of cytoplasmic vacuoles in MCF-7 cells (top and middle panels). Further, immunocytochemical analysis using an antibody against LC3 showed that 6-shogaol induced lipid modification of LC3-I into LC3-II, as characterized by the punctate localization of LC3 to autophagosomes in these cells (Fig 5, lower panel). Chloroquine (CQ), a drug approved by FDA, has been known to initiate massive autophagic response. But it inhibits the fusion of lysosomes to autophagosomes, thereby preventing autophagic progress and cell survival [29]. Chloroquine also induced cytoplasmic vacuoles in MCF-7 cells (Fig 5, top and middle panel) and massive punctuation of LC3 in the autophagosomes (Fig 5, lower panel).

However, increase in LC3-II signal may arise due to either increase in autophagic flux or due to defects in the downstream process of fusion of autophagosome and lysosome thereby inhibiting autophagic processing and degradation of LC3-II, as is seen in the cases of autophagic inhibitors chloroquine or baffilomycin.

**Fig 6:**
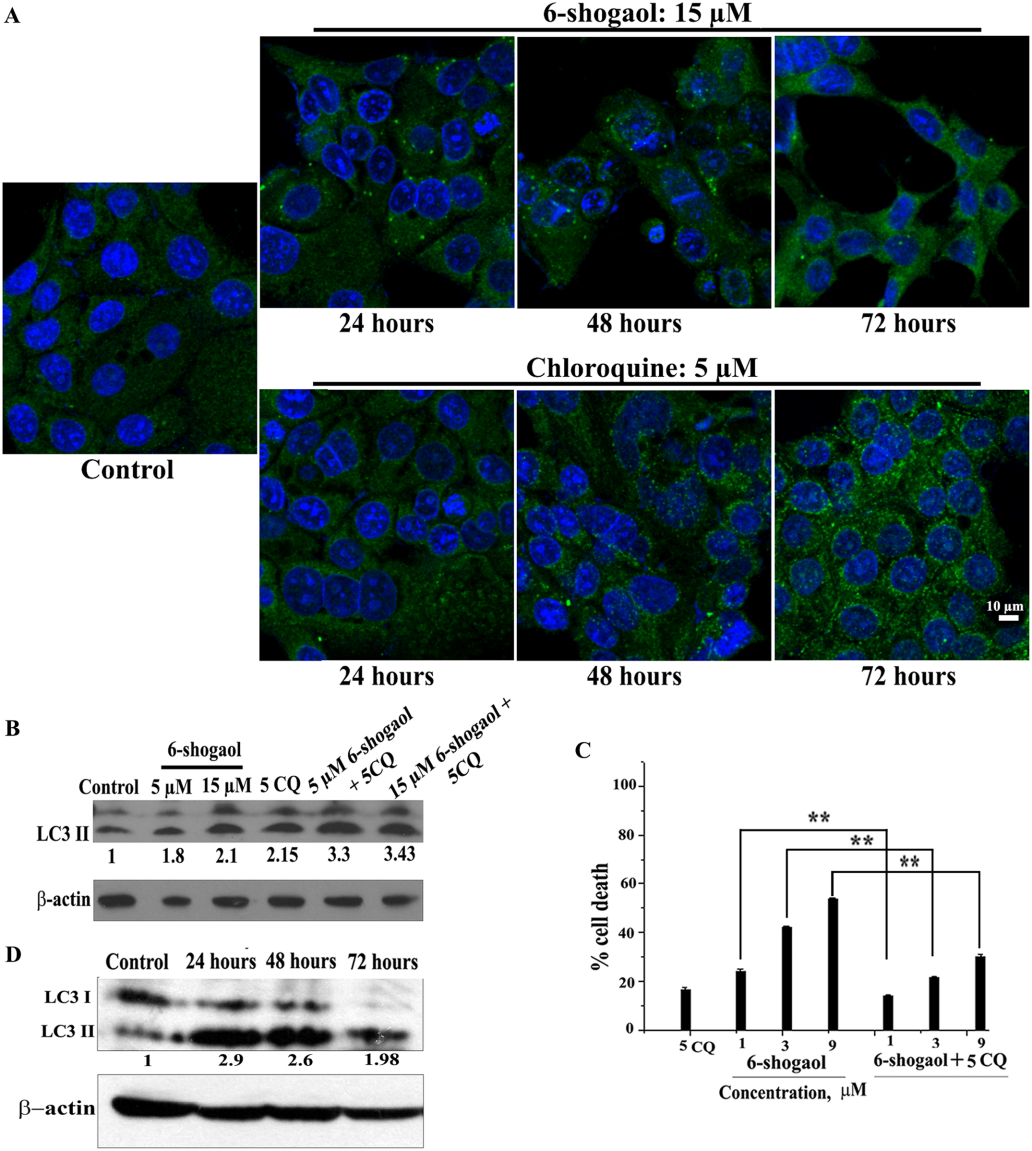
6-shogaol induces autophagic flux and cell death in breast cancer cells. (A): MCF7 cells were treated with 6-shogaol or chloroquine for the indicated time points, stained with LC3A/B antibody and imaged as described in the Methods section. Green punctae are indicative of LC3II expression. Cell nuclei are labelled in blue. (B): MCF7 cells were treated for 48 hours with the indicated concentrations of 6-shogaol or chloroquine or their combination. Western blot was conducted with 50 μg of protein. Fold change in the expression of LC3II was calculated with respect to β-actin level of the control. A representative blot has been shown from three independent experiments. (C): MCF-7 cells were treated with chloroquine (CQ), 6-shogaol or combination of CQ and 6-shogaol in the indicated concentrations. After 48 hours, cell viability was checked by MTT assay. The error bars represent the standard deviation from three different experiments. ** denotes p ≤ 0.005. (D): Western blotting was conducted with cell lysates of MCF-7 spheroids after 40 μM 6-shogaol treatment as detailed in the Methods section. Cleavage of LC3I to LC3II was observed in the MCF-7 spheroid cells. Housekeeping gene β-actin was used as loading control. LC3II expression was quantitated and fold increase with respect to the control has been indicated.

We examined whether 6-shogaol increased autophagic flux or induced any defect in autophagic processing after autophagosome formation in two ways. Immunofluoresence experiments at different time points detected increase in LC3 punctae upto 48 hours followed by decrease at 72 hours in case of 6-shogaol treatment. In contrast, continuous increase in LC3 accumulation with time was observed in case of chloroquine, indicating that unlike chloroquine treatment, LC3 got processed with time for 6-shogaol treatment (Fig 6A). Further, we used a low concentration of chloroquine in combination with 5 and 15 μM of 6-shogaol and checked the effect on LC3-II accumulation by western blotting. Since chloroquine is an autophagy inhibitor, an increase in LC3 content in the combination compared to 6-shogaol alone would indicate that autophagic flux generated by 6-shogaol is inhibited by chloroquine. We indeed found a significant increase in LC3-II expression in the combination of 5 and 15 μM of 6-shogaol with chloroquine (3.3 and 3.43 fold increase) when compared with the treatment of 6-shogaol alone (1.8 and 2.1 fold) (Fig 6B).

Further, to check the role of autophagy in the cell death induced by 6-shogaol, we used the autophagy inhibitor chloroquine, in a low concentration that limited the cell death induced by chloroquine itself. 5 μM chloroquine alone or in combination with 6-shogaol (1–9 μM) was added to MCF-7 cells and kept for 48 hours prior to analysis of the cell viability by MTT assay. Combination of chloroquine and 6-shogaol decreased the cell death percentage drastically from that of the cells treated with 6-shogaol alone (Fig 6C). The decrease in cell death percentage correlated with the increase in LC3 in combinations, indicating that cell death induced by 6-shogaol is dependent on the completion of autophagy.

Thus the results indicate that autophagy is a prominent mode of cell death triggered by 6-shogaol, although a modest amount of apoptosis was found in the cells after prolonged 6-shogaol treatment.

LC-3 cleavage upon 6-shogaol treatment was also observed in MCF-7 spheroids (Fig 6D). All these results imply that autophagy is induced by 6-shogaol treatment in breast cancer.

### Confirmation of 6-shogaol action on CSC like spheroid cells

The results so far indicated an inhibitory action of 6-shogaol on both breast cancer monolayer cells and spheroids. However, a possibility still remains that in a spheroid culture, 6-shogaol might actually work on the population of cells which don’t have stem cell-like characteristics and all the effects depicted so far are due to its action on mass cancer cells without stem cell-like phenotype. To rule out this possibility, we checked the expression levels of cell surface markers of breast cancer spheroids in the presence and absence of 6-shogaol. We also verified the effect of 6-shogaol on secondary sphere formation, a property which is strictly exhibited by cancer stem cell-like cells.

**Fig 7:**
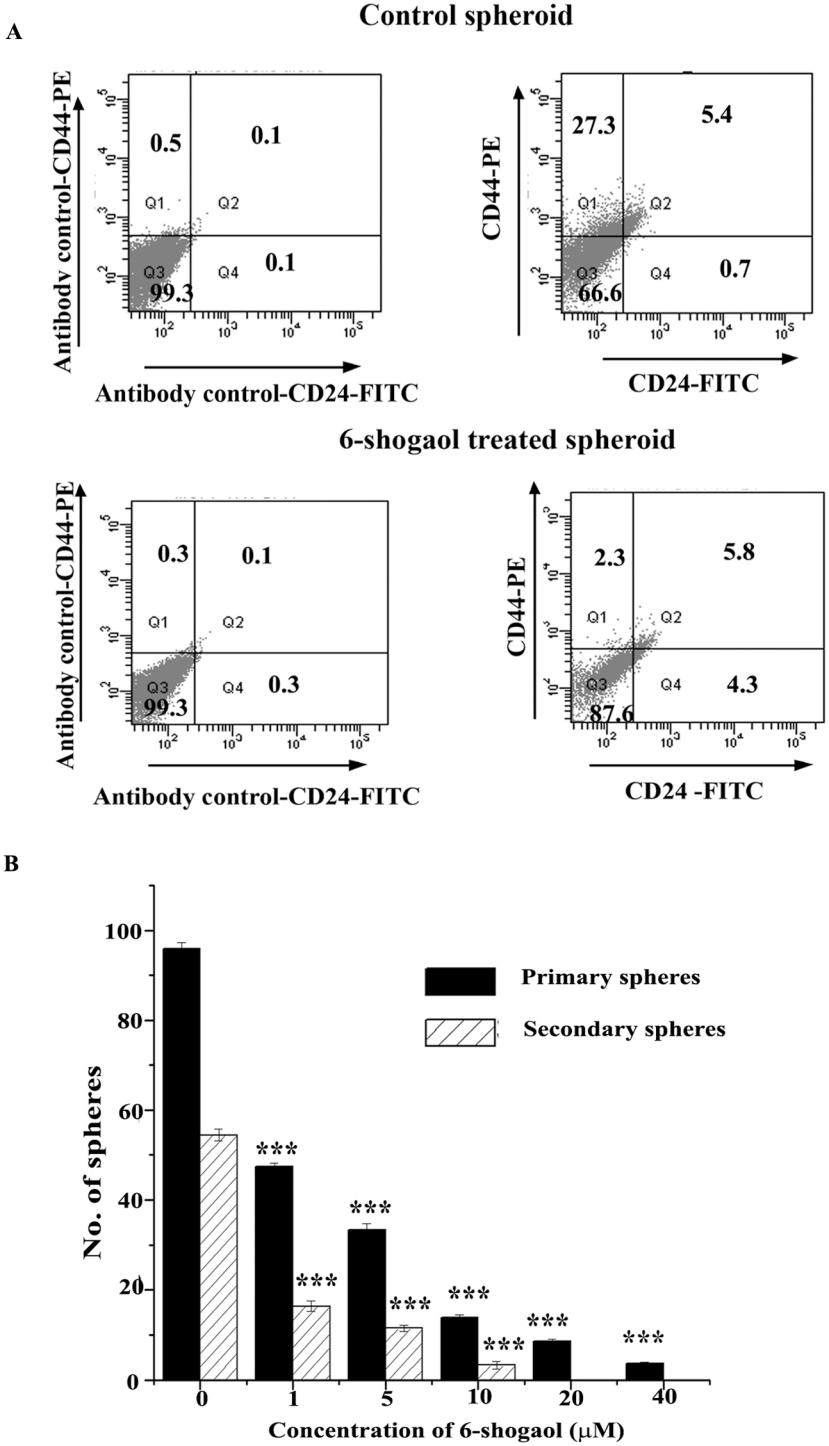
Confirmation of the action of 6-shogaol on breast cancer spheroids. (A): Flow cytometric analysis of CD44^+^ /CD24^-^ expression in untreated spheres (right upper panel) and spheres treated with 40 μM 6-shogaol for 18 hours (right lower panel). (B): Effect of different concentrations of 6-shogaol on primary and secondary spheres. 5000 cells per well were seeded in quadruplicates with or without 6-shogaol. After 7 days, number of spheres were counted and then dispersed. From these, 5000 cells were again seeded and kept to regrow in fresh media without 6-shogaol. The error bars represent the standard error of mean from three different experiments. *** refers p ≤ 0.001.

Flow cytometric analysis of breast cancer spheroids treated with 40 μM 6-shogaol for 18 hours (Fig 7A) showed that the percentage of cells characterized by CD44^+^CD24^-^/^low^ were substantially less in the treated spheroids when compared to the untreated ones (2.3% vs. 27.3%).

We next examined whether 6-shogaol was able to suppress the number and size of the spheres and if the effect of the compound was exhibited in the next generation of the spheres also. The MCF-7 spheroids at their stable growth condition after 3 days of seeding were treated with 1–40 μM of 6-shogaol and after 7 days of continuous treatment, the number of spheroid colonies were counted for each 6-shogaol concentration. A drastic reduction in the number of spheroid colonies was observed in a concentration dependent way. For example, average 96 spheroid colonies were found in control wells in the absence of 6-shogaol, whereas the average number of colonies was reduced to 48 (2 fold) with the treatment of only 1 μM 6-shogaol. Only 4 colonies were observed under the treatment of 40 μM 6-shogaol resulting a 24 fold inhibition (Fig 7B).

Next, the ability of the primary spheroids to grow as the next generation spheroids (secondary spheroids) was checked. The primary spheroids remaining after 1 week of continuous treatment with 6-shogaol were disaggregated and allowed to grow in fresh media without 6-shogaol for another week. While the control (generated without 6-shogaol treatment) primary spheroid cells produced ~ 55 secondary spheroid colonies, 1, 5 and 10 μM 6-shogaol-treated primary colonies were able to generate only a few secondary colonies with 71%, 80% and 94.5% inhibition, respectively (Fig 7B). Not a single secondary sphere was found at concentrations above 10 μM of 6-shogaol, indicating that 6-shogaol completely abolished the regeneration ability of the spheroids. Taken together, the results demonstrate that 6-shogaol effectively inhibits breast cancer spheroids.

### Effect of 6-shogaol on Notch signaling pathway

Notch signaling is known to be actively involved in stem cell self-renewal. It has also been found to be deregulated in most solid cancers including breast cancer [30, 31]. Notch signaling is activated when notch ligands (Delta, Delta like, Jagged1 and Jagged2) from one cell bind to the notch transmembrane (Notch1 to Notch4) receptor on the adjacent cells [30, 32]. This process usually initiates the γ-secretase mediated proteolytic cleavage of the Notch intracellular domain (NICD) which then translocates into the nucleus [30] and activates variety of Notch target genes such as Hes1 (Hairy enhancer of split) and Cyclin D1.

**Fig 8:**
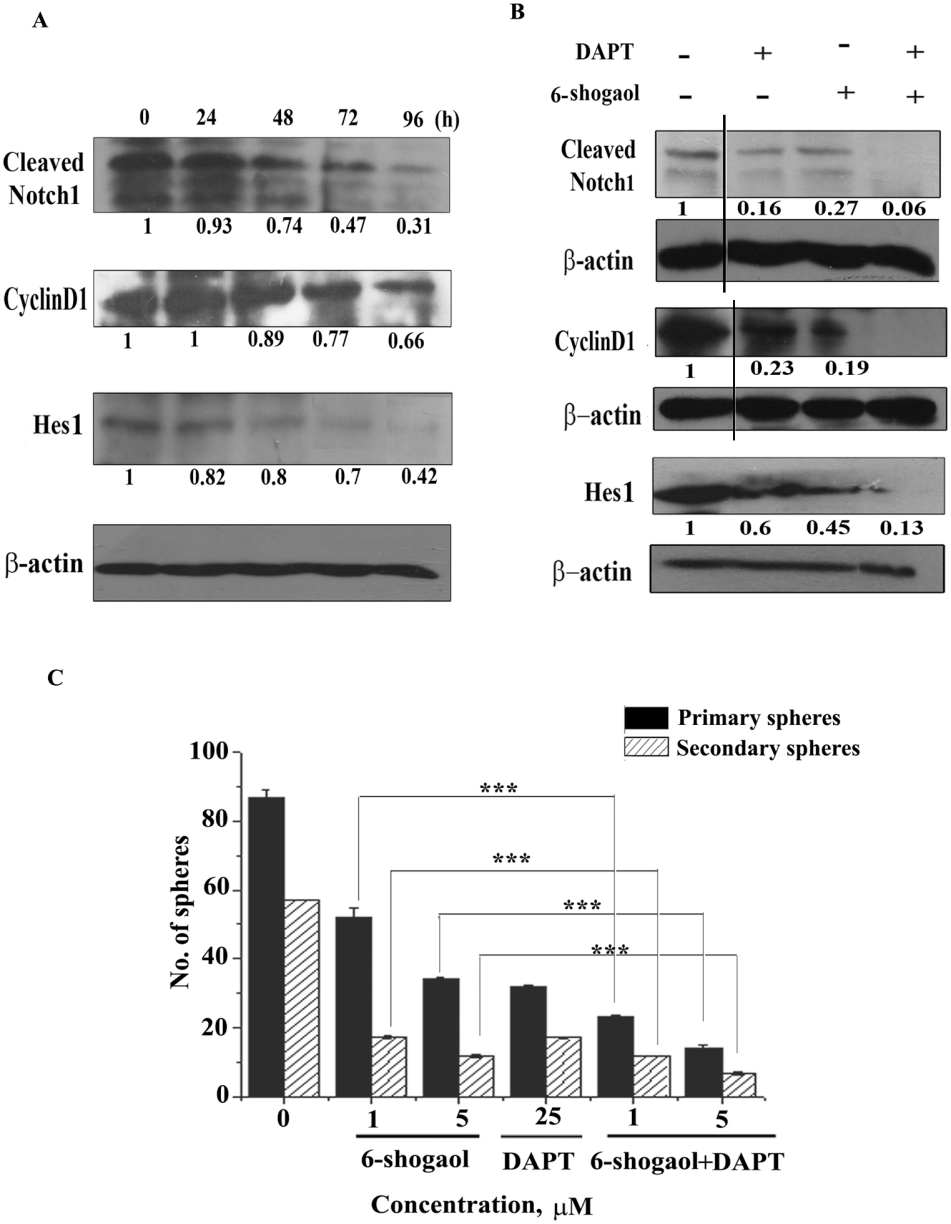
Modulation of Notch1 signaling pathway by 6-shogaol in MCF-7 spheroids. (A): Expression of Cleaved Notch1 and its targets Cyclin D1 and Hes1 after the treatment of 25 μM of 6-shogaol for the indicated time periods. (B): Expression of Cleaved Notch1, Cyclin D1 and Hes1 after 24 hour treatment of 6-shogaol in presence of the γ-secretase inhibitor DAPT (50 μM). The vertical lines in the Cleaved Notch1, Cyclin D1 and corresponding actin blots are to show a discontinuation to remove the lanes for 16 hours treatments that do not show any change in the protein levels. All the blots are representative of three independent experiments. (C): Effect of DAPT (25 μM) on the inhibition of primary and secondary sphere formation by 6-shogaol. The error bars represent the standard error of mean from three different experiments. *** denotes p ≤ 0.001.

To check whether Notch pathway is involved in 6-shogaol-mediated suppression of spheroids, the spheroid cells were treated with 25 μM of 6-shogaol upto 4 days and checked for the possible interference of Notch pathway. Treatment of 6-shogaol (25 μM) significantly reduced the cleavage of Notch1 as evident from the time-dependent reduction of cleaved Notch1 (110 kDa) (Fig 8A). The expression levels of Notch targets, Hes1 (30 kDa) and Cyclin D1 (34 kDa) also gradually decreased with time. Further we have investigated the mechanism of inhibition of spheroids by 6-shogaol treatment using DAPT, a γ-secretase inhibitor. While both DAPT and 6-shogaol individually reduced the cleavage of Notch1 and down-regulated the Notch target proteins Hes1 and Cyclin D1, a combination of DAPT and 6-shogaol had an additive effect and exhibited further reduction in the expression of these proteins (Fig 8B).

The effect of DAPT on formation of primary and secondary spheres was also checked. As shown in Fig 8C, the number of primary spheres was reduced to similar extents by individual treatments of 25 μM DAPT and 5 μM 6-shogaol. However, 6-shogaol was somewhat more effective in reducing the secondary spheres compared to DAPT. The combined treatment of DAPT and 6-shogaol further reduced the number of spheres as compared with the individual treatments. The results indicate that 6-shogaol inhibits the breast cancer spheroid formation by altering Notch signaling pathway through γ-secretase inhibition.

## Discussion

Cancer stem cells pose serious obstacle to cancer therapy as they can be responsible for poor prognosis and tumour relapse. To add into the misery, very few chemotherapeutic compounds show promise to kill these cells. Several researchers have shown that cancer stem cells are resistant to paclitaxel, doxorubicin, 5-fluorouracil, and platinum drugs [8, 16]. CSCs are thus an almost unreachable population in tumours for chemotherapy. Therefore any compound, that shows promise towards cancer stem cells, is a highly desirable step towards cancer treatment and should be followed up for further development.

Dietary compounds are welcome options for human diseases due to their time-tested acceptability by human bodies. Curcumin [33], quercetine [34], garlic products [35], ginger products [36] are some of them which show very high potency in many human diseases including cancer. In this study we have found that the ginger product 6-shogaol was effective in breast cancer cells in monolayer culture and spheroid culture in comparable concentrations and in conditions where taxol, even though highly effective in monolayer cells, was completely ineffective (Table 1). The effective concentrations of 6-shogaol were also safe to noncancerous cells as shown from the significantly higher IC_50_ values in case of HEK 293 and HaCaT cells even after 6 days (Table 1 and Fig 2C). The breast cancer spheroids generated by us under specific culture conditions showed altered levels of the breast CSC markers CD44^+^CD24^-^/^low^. These cells also showed secondary sphere forming capacity. 6-shogaol could drastically reduce the percentage of cells expressing the markers as well as the secondary sphere formation showing that it really could target the cancer stem cell like cells.

Cell cycle analysis of 6-shogaol treated cells showed G2/M arrest in both monolayer and spheroids. This is expected as 6-shogaol has been shown to interact with microtubules causing a mitotic block [7]. However, the percentage of apoptotic cells induced in MCF-7 monolayer cells was rather low as determined by different apoptotic assays. Further, apoptosis occurred only by a 2–3 times higher IC_50_ concentration of 6-shogaol. In spheroid culture, we couldn’t see considerable apoptotic cells even after 96 hours at a concentration slightly higher than the IC_50_. However, massive cell death at that concentration and beyond indicated other mechanisms of cell death.

Autophagy is considered as a basic survival mechanism of cells under stress. However, many reports show that autophagy is induced with a death trigger. Debate is still going on whether autophagy is another cell death mechanism besides apoptosis or cells die with autophagy using it as an effort to survive [37]. Induction of autophagy in 6-shogaol treated breast cancer cells was proved by cytoplasmic vacuole formation as well as the recruitment and cleavage of the microtubule associated protein Light Chain3 (LC3). As apoptosis was found to be a minor mode of cell death in these cells, the role of autophagy in 6-shogaol treatment needed further perusal. Our kinetic study of LC3 accumulation as well as combination studies with the autophagy inhibitor chloroquine (CQ) for LC3 accumulation and cell death convincingly proved that autophagy was the predominant cell death mechanism induced by 6-shogaol in breast cancer cells. A former report also showed that 6-shogaol was able to cause autophagy in HNSCLC A-549 cells and autophagy was the death mechanism in those cells in absence of apoptosis [4].

Several earlier studies have shown that dietary compounds which are used as anticancer agents, exhibited their interference with self-renewal pathways. Sulforaphane, curcumin and piperine inhibited Wnt pathway in breast cancer stem cells [14, 17]. Curcumin showed Notch pathway inhibition in esophageal cancer cells [38]. Notch signaling plays an important role in the development of mammary gland and is activated in stem cell self-renewal and differentiation [12, 39]. Many solid tumours including breast tumour also show deregulated Notch signaling [30, 40, 41]. 6-shogaol was found to interfere with the Notch pathway. Further, in our study, combination of the γ-secretase inhibitor DAPT and 6-shogaol resulted in further reduction of the expression of Notch and its target proteins (Fig 8B) when compared to the reduction by either DAPT or 6-shogaol alone. The combination also gave rise to lesser number of primary and secondary spheres than those generated from the individual treatments (Fig 8C). This indicates that even though both DAPT and 6-shogaol inhibited γ-secretase, their action was not competitive. γ-secretase mediated downregulation of Notch signaling followed by reduction in Notch1 downstream products like Hes1 and cyclin D1 by 6-shogaol thus explain its efficacy in breast cancer spheroid cells.

An earlier pharmacokinetic study [42] of 6-shogaol and three gingerols on healthy humans reported that after p.o. (orally administered) dosing, all these compounds were absorbed. These compounds were later found to be metabolized as glucuronide and sulfate conjugates. No serious adverse effects were seen on those persons after ingestion of a single dose of 2 g of standardized ginger extract. A former report also showed that 6-shogaol was not toxic to normal cells like MRC5, NP69, HaCaT and HMEC [6]. Our results on immortalized noncancerous cells thus supported that 6-shogaol did not have potent cytotoxicity to normal cells.

**Fig 9:**
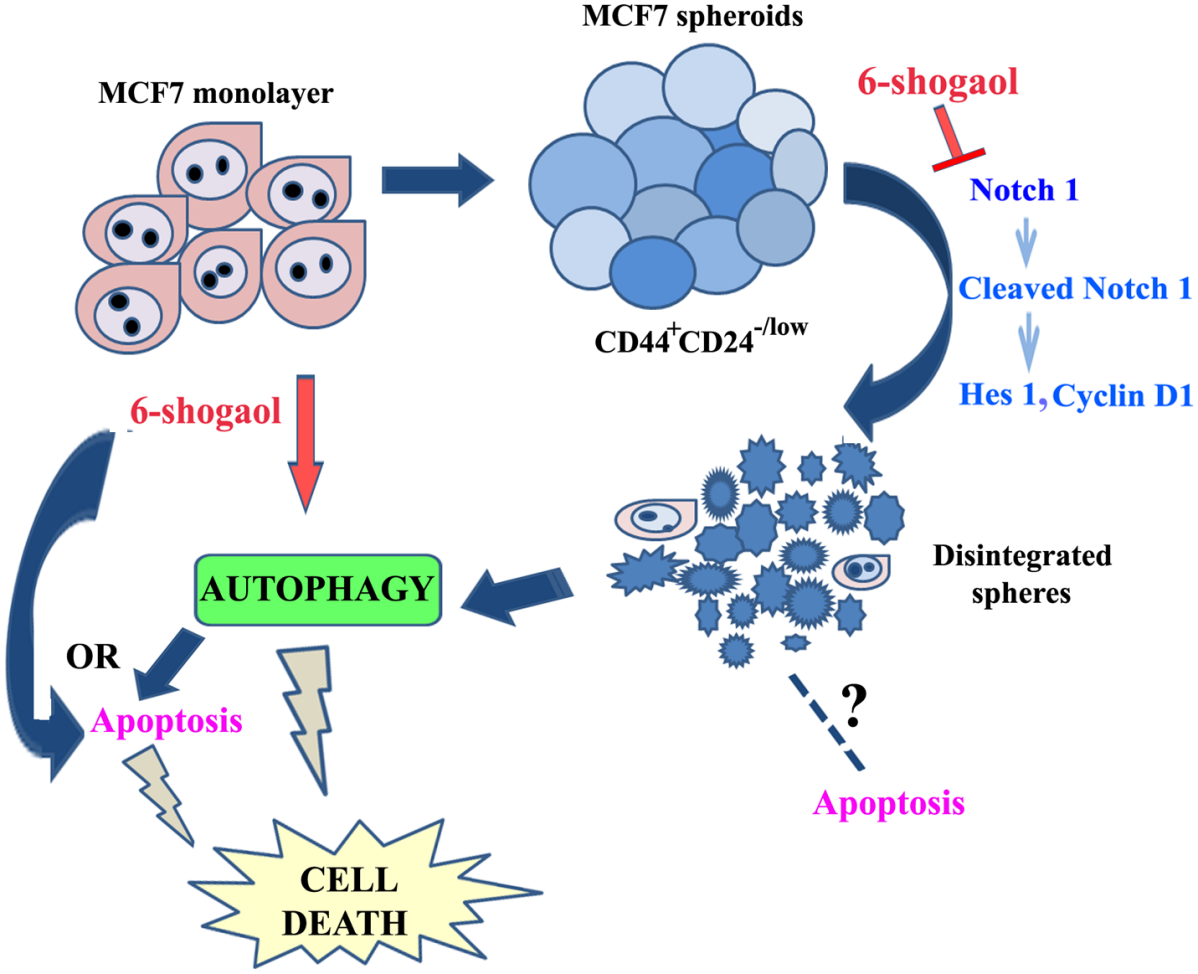
A schematic diagram showing the action of 6-shogaol on breast cancer cells and spheroids.

Our study shows the efficacy of 6-shogaol on both breast cancer monolayer cells and spheroids (a scheme shown in Fig 9) by interfering with stem cell self-renewal pathway. This report thus places it as a promising therapeutic agent which should be further followed up for breast cancer treatment.

## Supporting Information

S1 Fig Limited induction of apoptosis by 6-shogaol treatment for different time periods on MCF-7 cells. (A) Annexin V-FITC flow cytometry profile; (B) Cleavage of PARP by Western blot probed with CST-9544 antibody.
(TIF)

S2 Fig Full Length Blot for Fig 4D and 4E. (TIF)

S3 Fig Full Length Blot for Fig 6B. (TIF)

S4 Fig Full Length Blot for Fig 6D. (TIF)

S5 Fig Full Length Blot for Fig 8A. (TIF)

S6 Fig Full Length Blot for Fig 8B. (TIF)
