# Supplementary figures and images for: 6-Shogaol Inhibits Breast Cancer Cells and Stem Cell-Like Spheroids by Modulation of Notch Signaling Pathway and Induction of Autophagic Cell Death
Source: PLoS One. 2015 Sep 10;10(9):e0137614. doi: 10.1371/journal.pone.0137614 (PMC4565635; doi:10.1371/journal.pone.0137614)

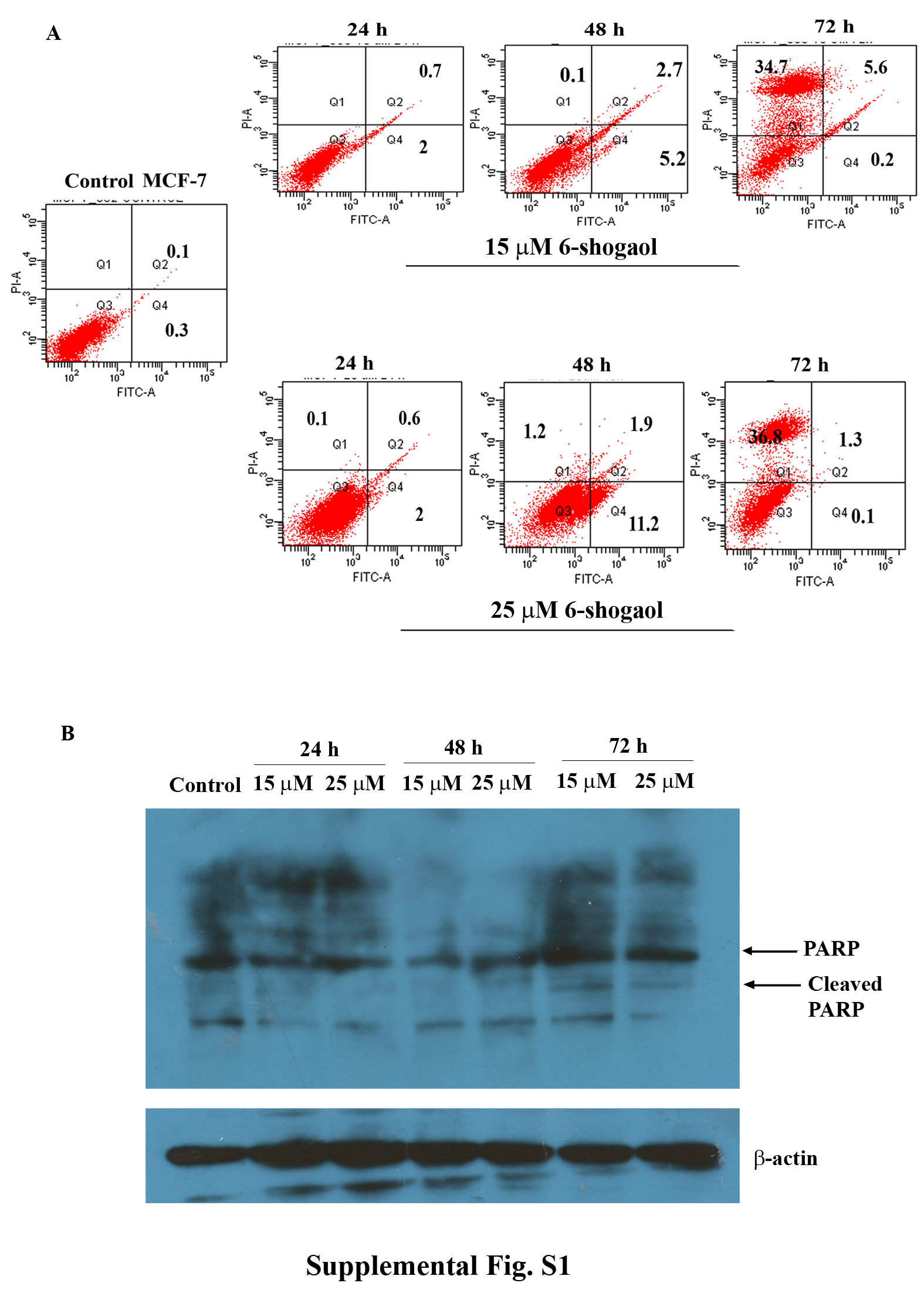

Supplement: S1 Fig — (A) Annexin V-FITC flow cytometry profile; (B) Cleavage of PARP by Western blot probed with CST-9544 antibody. (TIF) [file pone.0137614.s001.tif]

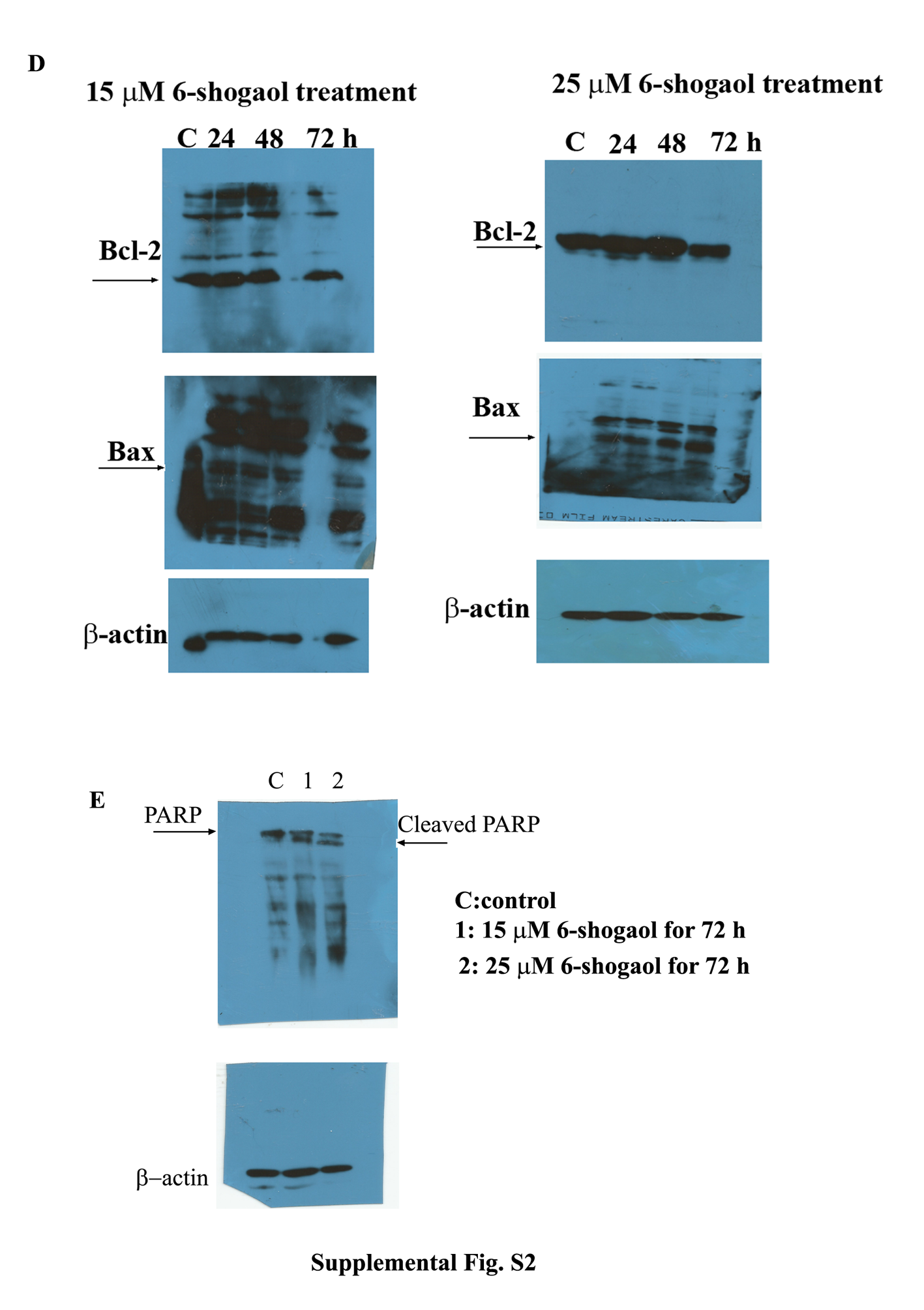

Supplement: S2 Fig — (TIF) [file pone.0137614.s002.tif]

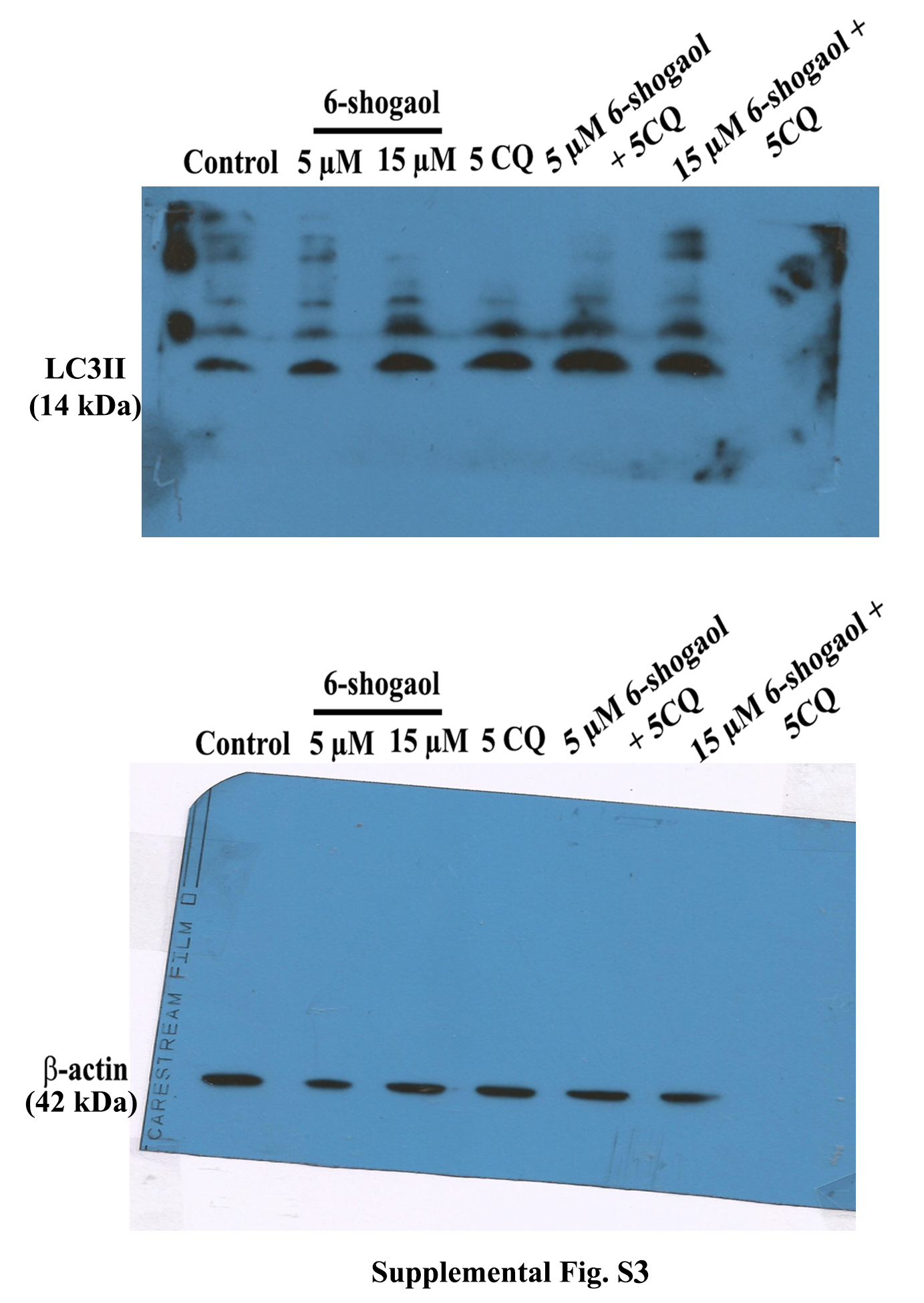

Supplement: S3 Fig — (TIF) [file pone.0137614.s003.tif]

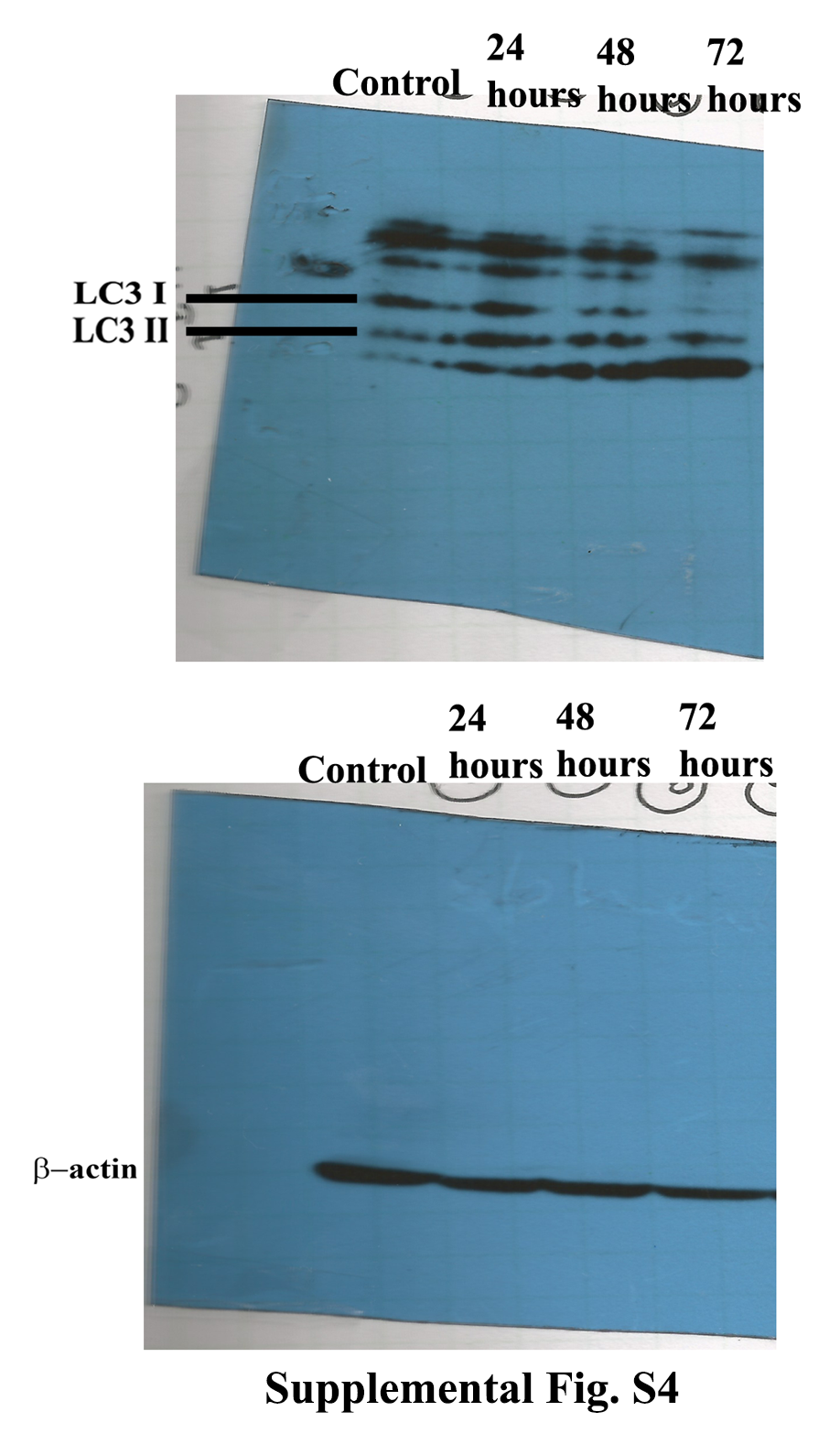

Supplement: S4 Fig — (TIF) [file pone.0137614.s004.tif]

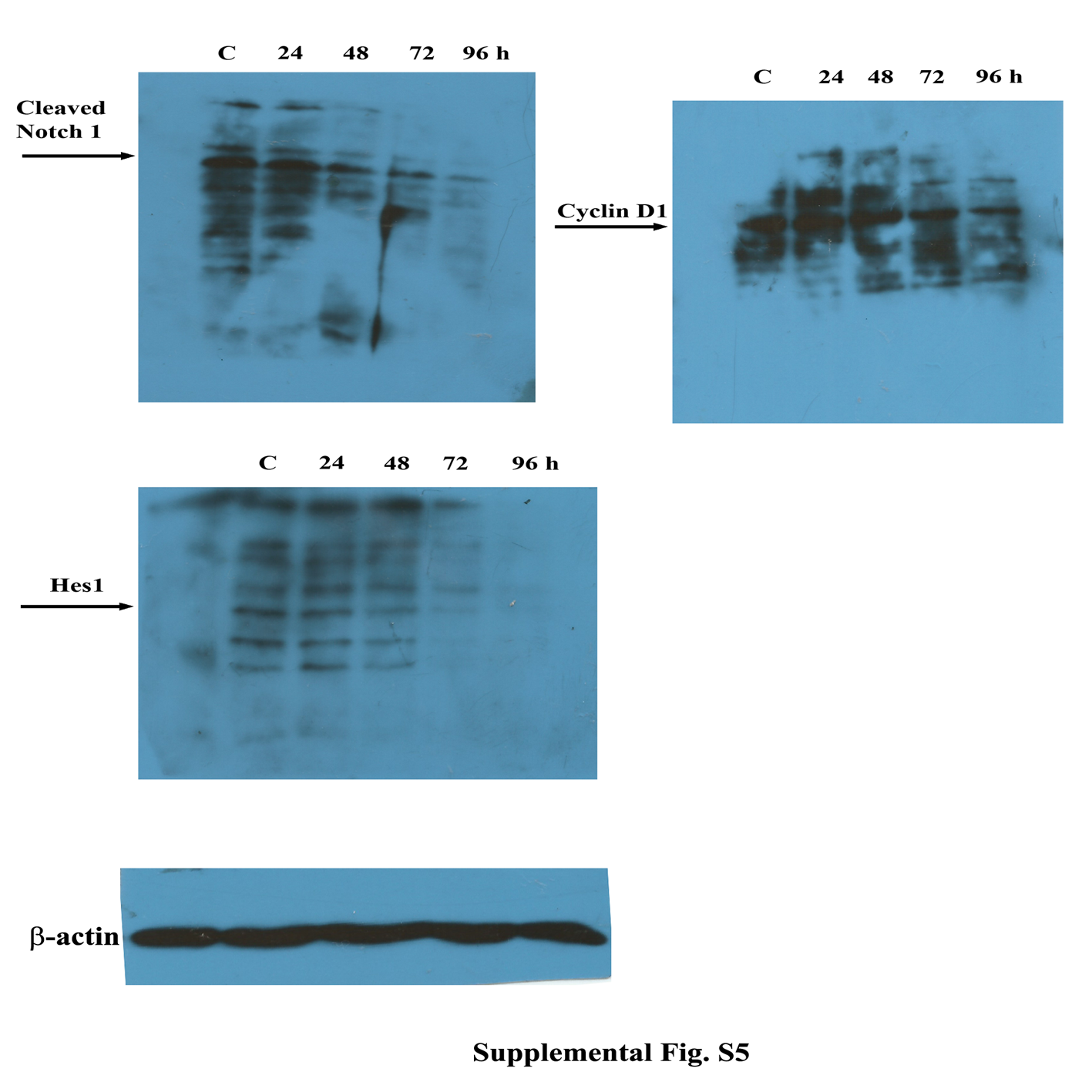

Supplement: S5 Fig — (TIF) [file pone.0137614.s005.tif]

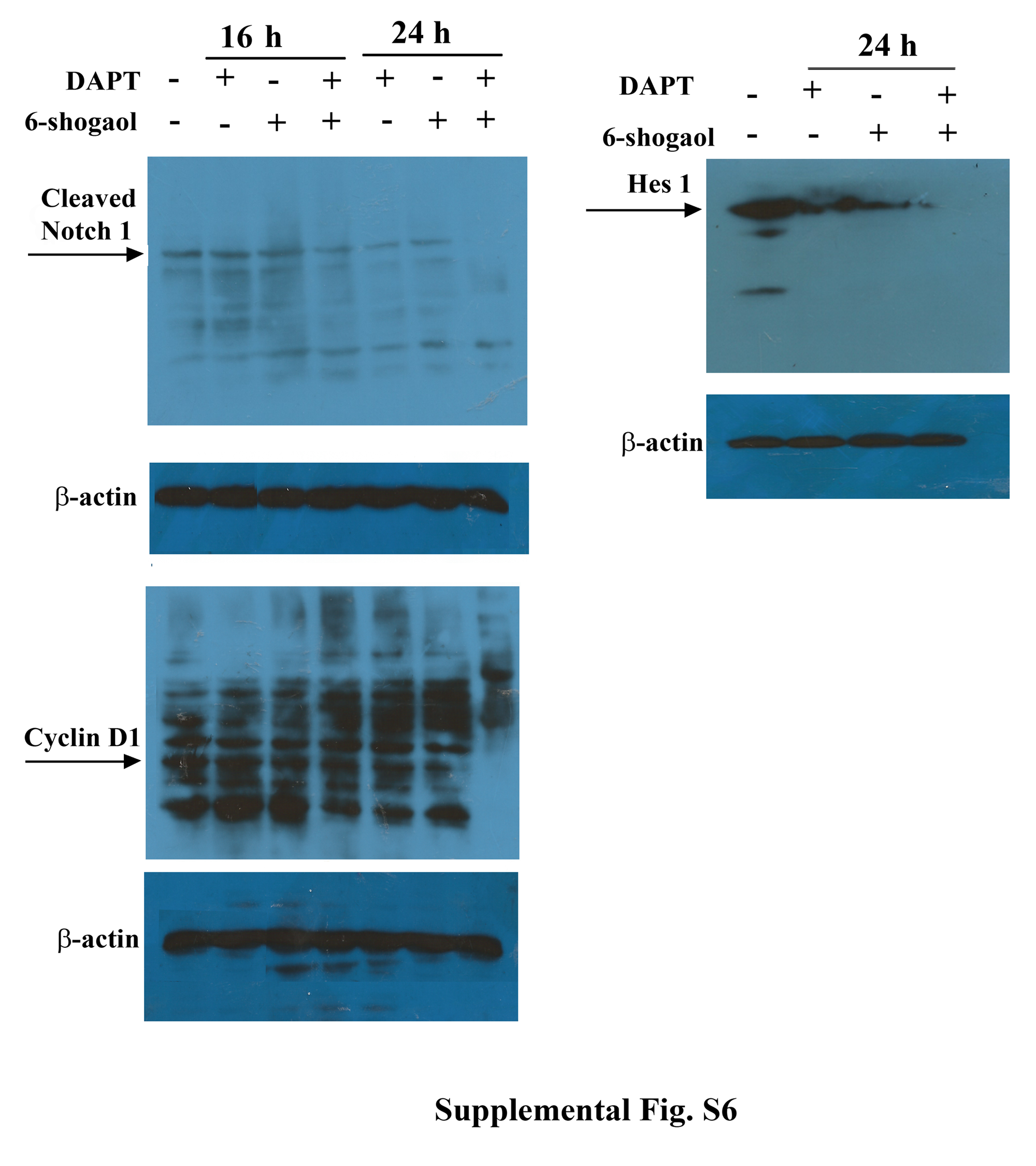

Supplement: S6 Fig — (TIF) [file pone.0137614.s006.tif]
